# Evaluation of Hybrid VMAT Advantages and Robustness Considering Setup Errors Using Surface Guided Dose Accumulation for Internal Lymph Mammary Nodes Irradiation of Postmastectomy Radiotherapy

**DOI:** 10.3389/fonc.2022.907181

**Published:** 2022-07-22

**Authors:** Zhe Zhang, Daming Li, Feng Peng, Zhibo Tan, Pengfei Yang, Zhaoming Peng, Xin Li, Xinyue Qi, Weixiao Sun, Yajie Liu, Yuenan Wang

**Affiliations:** ^1^ Department of Radiation Oncology, Peking University Shenzhen Hospital, Shenzhen, China; ^2^ Hong Kong University of Science and Technology Medical Center, Shenzhen-Peking University, Shenzhen, China; ^3^ Department of Statistics and Data Science, Southern University of Science and Technology, Shenzhen, China

**Keywords:** SGRT, H-VMAT, PMRT, IMNIs, biological models, setup error

## Abstract

**Objectives:**

Setup error is a key factor affecting postmastectomy radiotherapy (PMRT) and irradiation of the internal mammary lymph nodes is the most investigated aspect for PMRT patients. In this study, we evaluated the robustness, radiobiological, and dosimetric benefits of the hybrid volumetric modulated arc therapy (H-VMAT) planning technique based on the setup error in dose accumulation using a surface-guided system for radiation therapy.

**Methods:**

We retrospectively selected 32 patients treated by a radiation oncologist and evaluated the clinical target volume (CTV), including internal lymph node irradiation (IMNIs), and considered the planning target volume (PTV) margin to be 5 mm. Three different planning techniques were evaluated: tangential-VMAT (T-VMAT), intensity-modulated radiation therapy (IMRT), and H-VMAT. The interfraction and intrafraction setup errors were analyzed in each field and the accumulated dose was evaluated as the patients underwent daily surface-guided monitoring. These parameters were included while evaluating CTV coverage, the dose required for the left anterior descending artery (LAD) and the left ventricle (LV), the normal tissue complication probability (NTCP) for the heart and lungs, and the second cancer complication probability (SCCP) for contralateral breast (CB).

**Results:**

When the setup error was accounted for dose accumulation, T-VMAT (95.51%) and H-VMAT (95.48%) had a higher CTV coverage than IMRT (91.25%). In the NTCP for the heart, H-VMAT (0.04%) was higher than T-VMAT (0.01%) and lower than IMRT (0.2%). However, the SCCP (1.05%) of CB using H-VMAT was lower than that using T-VMAT (2%) as well as delivery efficiency. And T-VMAT (3.72) and IMRT (10.5).had higher plan complexity than H-VMAT (3.71).

**Conclusions:**

In this study, based on the dose accumulation of setup error for patients with left-sided PMRT with IMNI, we found that the H-VMAT technique was superior for achieving an optimum balance between target coverage, OAR dose, complication probability, plan robustness, and complexity.

## Introduction

Radiation therapy is an integral part of the comprehensive treatment of breast cancer and has significantly improved the overall survival rate of breast cancer (1–6). But for left-sided breast cancer including internal lymph mammary nodes irradiation (IMNIs), the protection of the organs at risk (OARs) has always been the focus of discussion. An increase in cardiac, especially for the left anterior descending artery (LAD), significantly increases the incidence of ischemic heart disease (1–3). In a study, Darby found that for every 1Gy increase in the mean heart dose, the risk of coronary heart disease increases by 7.4% (1). For women receiving breast radiation therapy, the radiation pneumonitis (RP) of the ipsilateral lung is higher than that of the contralateral lung (4, 5). Fogliata (6) found that for young breast cancer patients, the radiation dose used for treating the contralateral breast (CB) might lead to long-term risks, and the incidence of secondary tumors is also affected by the dose received by the CB. Various techniques, including tangential-VMAT (T-VMAT), intensity-modulated radiation therapy (IMRT), and Hybrid-VMAT (H-VMAT) can reduce the dose of surrounding OARs in modern radiotherapy (7–9).

To determine the dose distribution of the target volume and the OARs during treatment, the setup error needs to be considered (10). Some of the methods used for evaluating the setup errors are based on the value obtained by performing CBCT (11), but these methods may not pay much attention to the interfraction setup error. In this study, we performed surface guided monitoring to obtain the intrafraction and interfraction setup error for analysis (12, 13), and then dose accumulation is performed to obtain a dose distribution for evaluating the robustness of all planning techniques. In some studies, dosimetry for left-sided breast cancer PMRT patients was compared to different planning techniques under dose distributions using setup uncertainty (12, 13), focusing on the evaluation of IMN included left-sided breast cancer based on biological models.

Furthermore, some studies have found that the parameters of these biological models can predict the effects of normal tissues (14–16). Compared to the parameters based on dosimetry, the parameters based on biological models are more directly related to complications and treatment endpoint events (17). This study also retrospectively compared three planning techniques associated with radiobiological effects, including the normal tissue complication probability (NTCP) and the second cancer complication probability (SCCP), considering setup error dose accumulation.

## Method

### Patient Selection

We selected 32 PMRT patients in the radiation therapy department at Peking University Shenzhen Hospital fromApril 2020 to September 2021 [**Table 1**]. The inclusion criteria were as follows: (1) Female patients over 18 years of age with left breast cancer who underwent PMRT; (2) Invasive diagnosis of cancer was confirmed by pathology; (3) Surgical margins were negative; (4) Who received chemotherapy and following pre-radiotherapy standards and guidelines.

**Table 1:** Patient characteristics.

Characteristic	Value
Age (years)	Median 49.5
	Range 30-65
Histologic grading (n)	Grade 2 14
	Grade 3 18
Tumor size (cm)	Median 3.25
	Range 1.5-10
ER/PR status (n)	Negative 13
	Positive 19
Her-2 status (n)	Negative 18
	Positive 14

### Treatment Planning Design

Free-breathing CT scan was performed from the level of the mandible to the lower abdomen on the SOMATOM Definition AS CT Scanner (Siemens Medical Solutions, Erlangen, Germany) with a slice thickness of 3 mm. The patients were immobilized on a customized vacuum bag in the supine position with arms placed above the head. The clinical target volume (CTV) and OARs for each patient were contoured by one radiation oncologist following the RTOG-1304 (18) guidelines and the RTOG Breast Cancer Atlas (18). CTV included the chest wall (CW), internal lymph mammary nodes (IMNs), and the axillary and supraclavicular lymph nodes. A 5 mm margin was added to the CTV to define as PTV, and the part that intersects the lung and heart was subtracted from the chest wall, but the 5 mm external expansion of IMNs was maintained (19). It is necessary to treat PMRT patients 10 times without bolus and 15 times with bolus in the treatment. However, in this study, only 25 times with bolus plan were evaluated. In the plan design, the PTV is expanded to the skin by 5 mm as an optimized condition for opening the MLC as much as possible. This was done following the procedure described in a study (20) to match the dose outside the skin boundary and reduce the impact of breathing motion on the skin dose.

The Eclipse software (TPS, Eclipse, version 15.6, Varian Medical Systems, Palo Alto, CA, USA) was used. In all plans, the prescribed dose was 50Gy/25 fractions with 6MV photons. The dose volume constraints on the TPS opitimization interface for planning target volume and OARs followed the same objective template [**Table 2**].

**Table 2:** The dose-volume constraints on TPS optimization interface for planning target volume and organs at risk.

PTV/OAR	Dose–volume constraints
PTV	D95%>5000 cGy
Heart	V20<15% D mean<800 cGy
LAD	D mean<3000 cGy
LV	D mean<1000 cGy
Ipsilateral Lung	V5<60% V20<25% V30<15% D mean<1400 cGy
Lungs	V5<60% V20<20% V30<10% D mean<800 cGy
Contralateral Lung	V5<60% D mean<600 cGy
Contralateral Breast	D0.1cc<2000 cGy D mean<500 cGy

**Figure 1:**
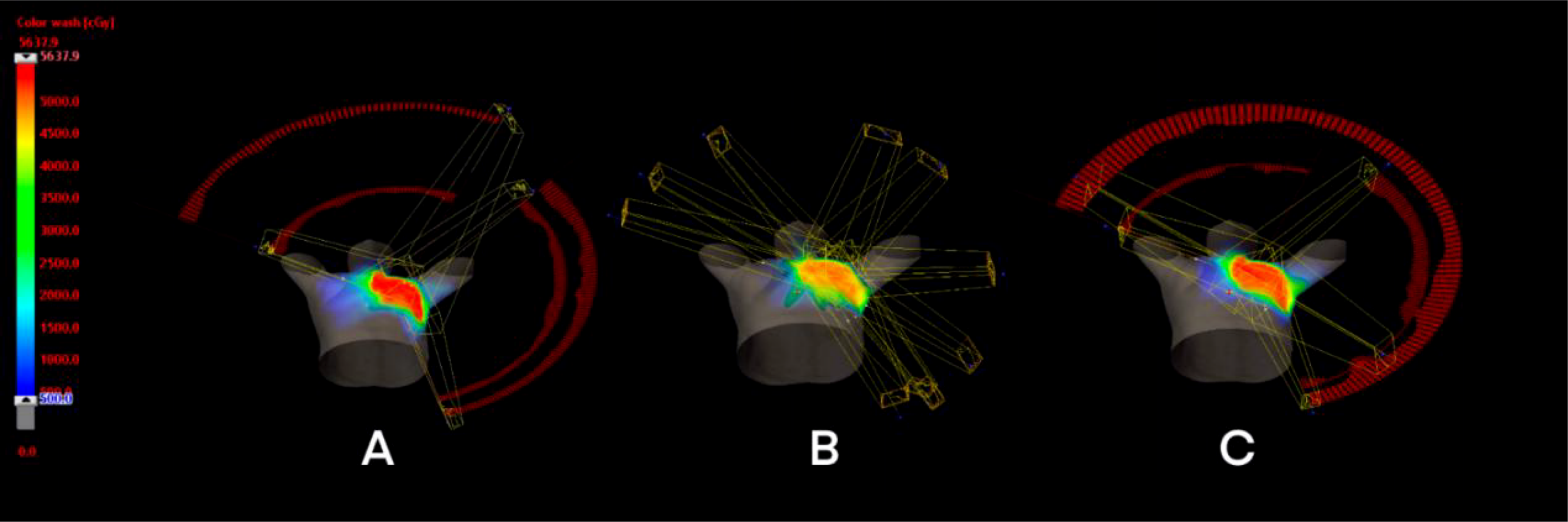
Treatment planning design for three techniques: **(A)** T-VMAT; **(B)** IMRT; **(C)** H-VMAT.

**Figure 2:**
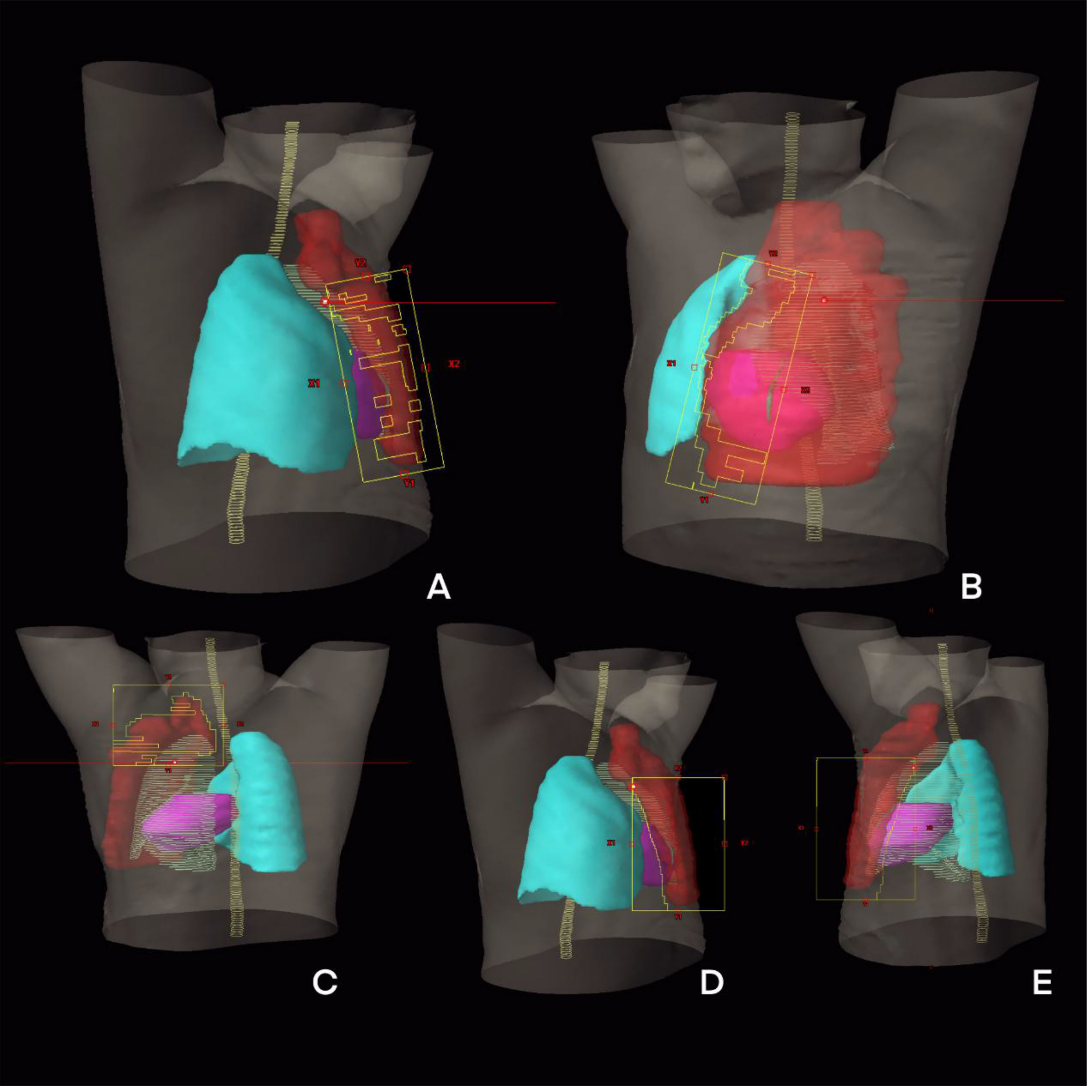
The beam eye view of treatment planning design of H-VMAT technique: **(A)** An arc from 295° to 20°; **(B)** An arc from 40° to 150°; **(C)** Tangential field one; **(D)** Tangential field two; **(E)** An arc from 150° to 295°.

The Tangential VMAT plan was designed as four partial arcs, where the upper and lower fields were connected from lymph node to chest wall. Arc 1 and Arc 2 are usually set to 295° to 20° and reversed, and Arc 3 and Arc 4 are set to 40° to 150° and reversed shown as **Figure 1A**. The IMRT plan contains 10 fields, of which three covered lymph nodes (20°, 40° and 160°), six covered chest wall PTV (290°, 315°, 340° and 90°, 120°, 150°) and one covered conjunction part (150°). The collimator irradiated the PTV at different angles while avoiding the lungs and the heart, and the dose outside the skin was compensated by brushing the fluence. To effectively protect the OARs, fixed jaw technology is used in all fields of vision [**Figure 1B**]. In addition, the hybrid VMAT in **Figure 1C** and **Figure 2** includes five fields, two tangential fields covering PTV-CW and IMNs at 70% dose, and two separate partial arcs covering approximately 30% of PTV-CW from 295° to 20° and 40° to 150°, an arc from 150° to 295° covered the PTV axillary and supraclavicular lymph nodes.

### Evaluating Setup Errors and Dose Accumulations

The retrospective study was performed with the Catalyst™ system (C-rad Positioning AB, Uppsala, Sweden). Data and surface images were collected every 50 mS for recording during the 25 fraction treatments in every PMRT patients, and the tolerance was set as 5 mm.

To obtain the Interfraction setup error for optical body surface monitoring, we performed surface acquisition at the first treatment after CBCT was performed as the reference, and then we collected surface data before the patients underwent IGRT. The non-rigid registration algorithm in the analysis tools in Catalyst™ was used to calculate any isocenter shift by matching the reference images and the images before IGRT. The region of interest (ROI) was set as the left-sided chest wall of the patients, which allowed the isocenter shifts to approximate the Interfraction setup error extraction for each treatment (12).

Every patient was treated using a specific treatment technique, and thus, it was impossible to obtain the three intrafraction setup errors in one patient. Hence, we selected 10 patients per technique (T-VMAT, H-VMAT, and IMRT) to estimate each intrafraction setup error during treatment. To derive the intrafraction setup error of the patients, we first retrieved data for each patient’s fraction, including isocenter shifts in the four degrees during the beam-on time, from Catalyst. The data of the 25 fractions for each patient was extracted and divided into five setup error sets, with each set representing the mean value of five fractions. Therefore, set 1 demonstrated the average setup error during the beam-on time from the first fraction to the fifth fraction. Furthermore, each field during the treatment had a slightly different setup error. We subdivided the intrafraction setup error for each field and combined the interfraction setup error; finally, the average setup error per set was calculated.

**Figure 3:**
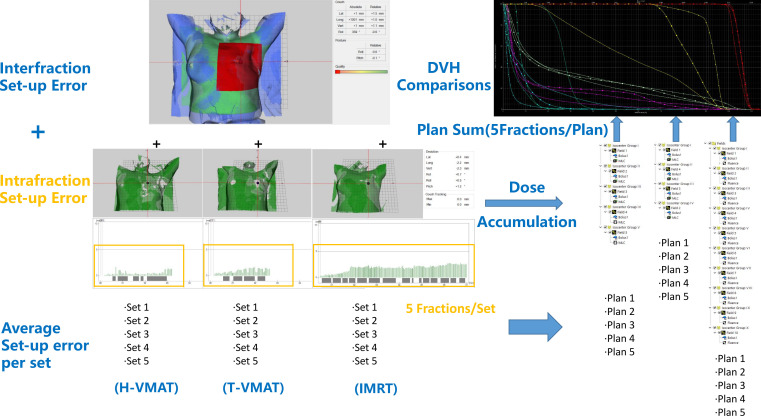
The workflow used to generate the SGRT-based setup errors and dose accumulation is shown. First, non-rigid algorithm was used for interfraction setup error analysis. Then we subdivided the intrafraction setup error of each field and each technique divided 25 fractions into 5 sets (5 fractions per set). Furthermore, sum up the average setup error for each set. Finally, the setup errors imported to TPS and converted each field into an isocenter group, then accumulated and compared dose. In the DVH comparisons figure, —shown as T-VMAT, —shown as H-VMAT, —shown as IMRT.

The setup error of each field in each set was obtained and imported into the Eclipse software to convert each field into an isocenter group. The setup error was entered in four degrees (longitudinal, transversal, vertical, and rotation) and re-calculated five times per technique for each patient. For example, in the H-VMAT plan, we initially used five fields. Next, we used five different isocenter groups for calculations in each set as plan 1 and the sum of five plans was evaluated for one patient using the H-VMAT technique. In total, 480 re-calculations were performed on five sets using each of the three techniques for the 32 patients. The re-calculations for each patient were followed by dose accumulation, and the resulting dose parameter and DVH are shown in **Figure 3**.

### Plan Evaluation

The dose parameters were read using the clinical protocol template on eclipse 15.5. The main dosimetry indicators include the coverage of CTV, the dose parameters of each OARs and the radiobiological indicators included lung and heart NTCP and CB SCCP. DVH was imported into MATLAB-based internal programs (MathWorks, Natick, MA) to calculate NTCP and SCCP values (14, 21). The pulmonary endpoint event was RP ≥ grade 2, which was calculated using the EUD-based NTCP model given by


(1) $$EUD={\left(\sum V_{i}\cdot D_{i}^{a}\right)}^{\frac{1}{a}}$$

Here, a is a unitless model parameter that is specific to the normal structure or tumor of interest, and V_i_ is unitless and represents the i’th partial volume receiving dose D_i_ in Gy.


(2) $$NTCP=\frac{1}{1+{\left(\frac{TD_{50}}{EUD}\right)}^{4\text{γ}50}}$$

The TD50 is the tolerance dose for a 50% complication rate at a specific time interval when the whole organ of interest is homogeneously irradiated (22), and the γ_50_ is a unitless model parameter that is specific to the normal structure or tumor of interest and describes the slope of the dose response curve The NTCP for lung calculation has the following parameters: TD_50_ = 24.5 Gy, a = 1, and γ_50 =_ 2 (21, 22).

The NTCP of heart used the NTCP-Poisson LQ function to calculate uses cardiac mortality as the end point (23). The dose-response curve for the complete organ volume is given by


(3) $$P\left(D\right)=2^{-\text{exp}\left\{e\text{γ}\left(1-D/D_{50}\right)\right\}}$$

Here, the dose for 50% response is denoted by D_50_ and the maximum relative slope of the dose-response curve is given by γ. In this model the organization of the functional subunit (FSU) is described in terms of a number of parallel strings in which each string consists of serially organized FSU. The relative seriality is given by the ratio of the number of serial subunits to all subunits and is described by the parameter *s.* For a heterogeneous dose distribution, the complication probability is determined by the equation


(4) $$P={\left\{1-\prod_{i=1}^{n}{\left[1-P{\left(D_{i}\right)}^{s}\right]}^{\Delta vi}\right\}}^{1/s}$$

Here, *n* is the number of subvolumes in the dose calculation volume (DVH), and Δv_i_= v_i_/V, where *v_i_* is the volume of each subvolume in the DVH and *V* is the volume of the organ. The parameters used in the model are D_50_ = 52.3 Gy, γ = 1.28, and s = 1 (20, 21).

Calculating the second cancer complication probability (SCCP) of the contralateral breast takes the secondary incidence of tumor as the endpoint event. The equation can be represented as


(5) $$SCCP_{org}=In_{org}\cdot \sum_{i}\left(V_{i}\cdot D_{i}\cdot e^{-\text{α}D_{i}}\right)$$

Here, α is the cell radio sensitivity (Gy^-1^) and In_org_ is the absolute cancer incidence rate in percent per gray for the specific organ. The parameters used for the calculation of the SCCP and Schneider model are α = 0.085 and In_org_ = 0.78%/Gy (22, 24).

To compare delivery efficiency and difficulty, the number of monitoring unit (MU), modulation factor and total delivery time were also quantitatively analyzed. The modulation factor is the total number of MU divided by the prescribed dose per fraction as follow. All data are expressed as the mean ± standard deviation. Wilcoxon signed-rank tests were performed in SPSS (25^th^ edition, Chicago, Illinois, USA) to determine significant differences (p < 0.05) between treatment planning techniques.


(6) $$MF\left(\frac{MU}{cGy}\right)=\frac{Total\;Plan\;Monitor\;Unit\left(MU\right)}{Prescribed\;Dose\left(cGy\right)}$$

## Results

### Dose Analysis

T-VMAT covered the largest percentage of CTV among the three methods (98.6%), and the differences between T-VMAT and the other two techniques were statistically significant (P ≤ 0.001); coverage was the lowest for the IMRT technique. After introducing the positioning error, the coverage rate of T-VMAT was still the highest (95.51%), but H-VMAT reached 95.48%. The difference between T-VMAT and H-VMAT was not significant (P = 0.428).

The mean heart dose (MHD) in T-VMAT was 5.34 Gy, which was the lowest dose among the three planning methods. In all pairwise comparisons showed significant differences (P ≤ 0.001). After dose accumulation, the MHD increased by different degrees, but T-VMAT had the lowest MHD, which was significantly different from IMRT and H-VMAT (P ≤ 0.001). The T-VMAT technique also had the lowest value for the mean dose of LV (5.76 Gy), but the difference in the values between H-VMAT and T-VMAT was not significant (P = 0.092). After re-calculation, the doses of all three groups increased, but the difference in the dose values between H-VMAT and T-VMAT was not significant (P = 0.871), while the p-values of the other two groups were significantly different (P ≤ 0.05). T-VMAT also had the lowest mean dose of LAD (28.9 Gy), and the difference between H-VMAT and T-VMAT was not statistically significant (P = 0.138). After adding the setup error, the mean dose of LAD for the three groups showed significant differences in pairwise comparisons (P ≤ 0.05).

Irrespective of whether the dose of the IL was V5, V20, V30, or the mean dose, IMRT was higher than the other two techniques. There was no significant difference in Dmean and V20, except for those of IMRT vs. H-VMAT. The other dose parameters showed significant differences in the pairwise comparisons (P ≤ 0.05). After introducing the positioning error, no statistical difference was found for the mean dose. For the whole lung, the highest V5 of the T-VMAT technique was 47.18%, the highest V20 of IMRT was 13.77%, and the highest Dmean of H-VMAT was 8.37 Gy. The results showed that only the five sets of data of IMRT compared to those of the other two techniques were significantly different (P ≤ 0.05), and there was no significant difference in the parameters between T-VMAT and H-VMAT. After re-calculation, the V5 and mean dose of the T-VMAT technique were the highest among the three techniques. V20 was the highest for the H-VMAT technique, but there was no significant difference when comparing V20 of IMRT with that of the other two groups (P = 0.247 with H-VMAT and P = 0.112 with T-VMAT).

**Figure 4:**
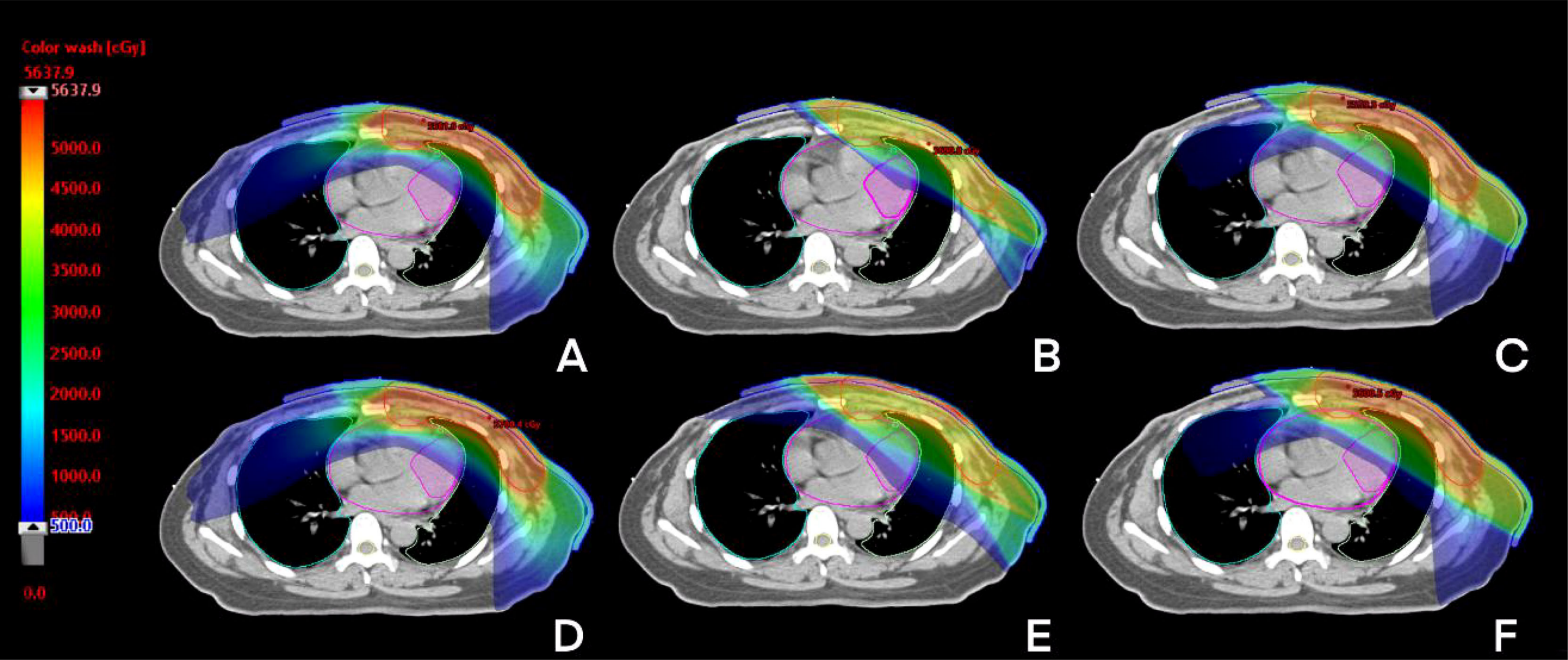
The dose distribution of three techniques before and after setup error re-calculation. A, B, C before setup error [T-VMAT **(A)**, IMRT **(B)**, H-VMAT **(C)**], **(D–F)** after setup error [T-VMAT **(D)**, IMRT **(E)**, H-VMAT**(F)**].

**Figure 5:**
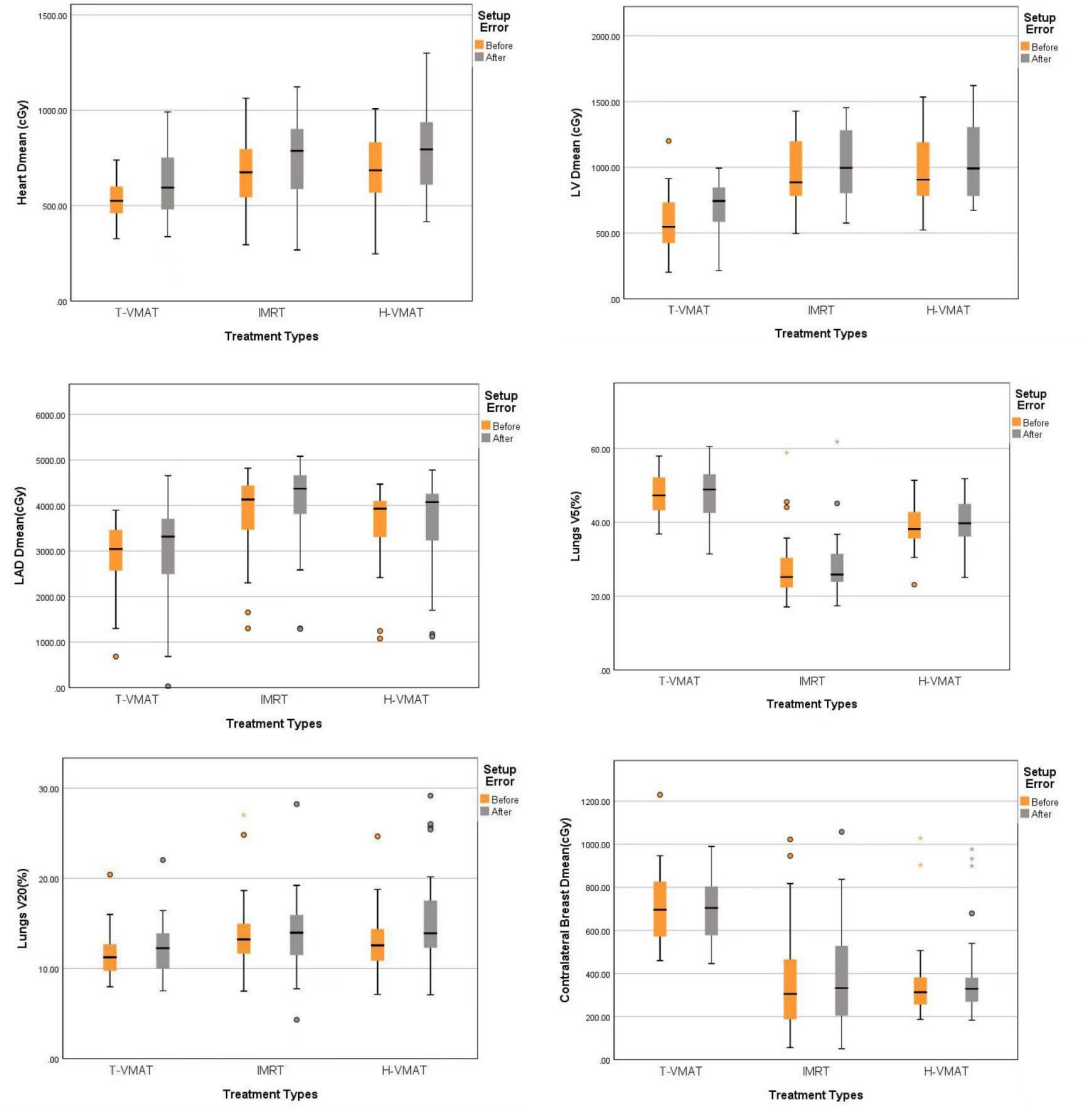
Box-whisker plot of dosimetry parameters with error bars:Heart Dmean, Left ventricle Dmean, Left anterior Dmean, Lungs V5, Lungs V20, Contralateral breast Dmean. Each figures show the dose changes of each OARs before and after the recalculation of setup error. Gray bars represent the accumulated dose after the consideration of setup error, and yellow bars represent the dose not considered of setup error. The points in the graph represent outliers, the black horizontal lines in the figures represent the average of each parameter.

For CB, all parameters of T-VMAT were significantly higher than those of IMRT and H-VMAT, and Dmean was 5.76 Gy. T-VMAT was significantly different from IMRT (P = 0.024), but T-VMAT was not significantly different from H-VMAT (P = 0.059). The average dose of T-VMAT after adding the setup error was 6.99 Gy. There was no significant difference between IMRT and H-VMAT (P = 0.334); however, the other two groups showed significant differences (P ≤ 0.05). The dose distribution and dosimetry data are shown in **Figures 4** and **5**, respectively.

### Biological Model Analysis

**Figure 6:**
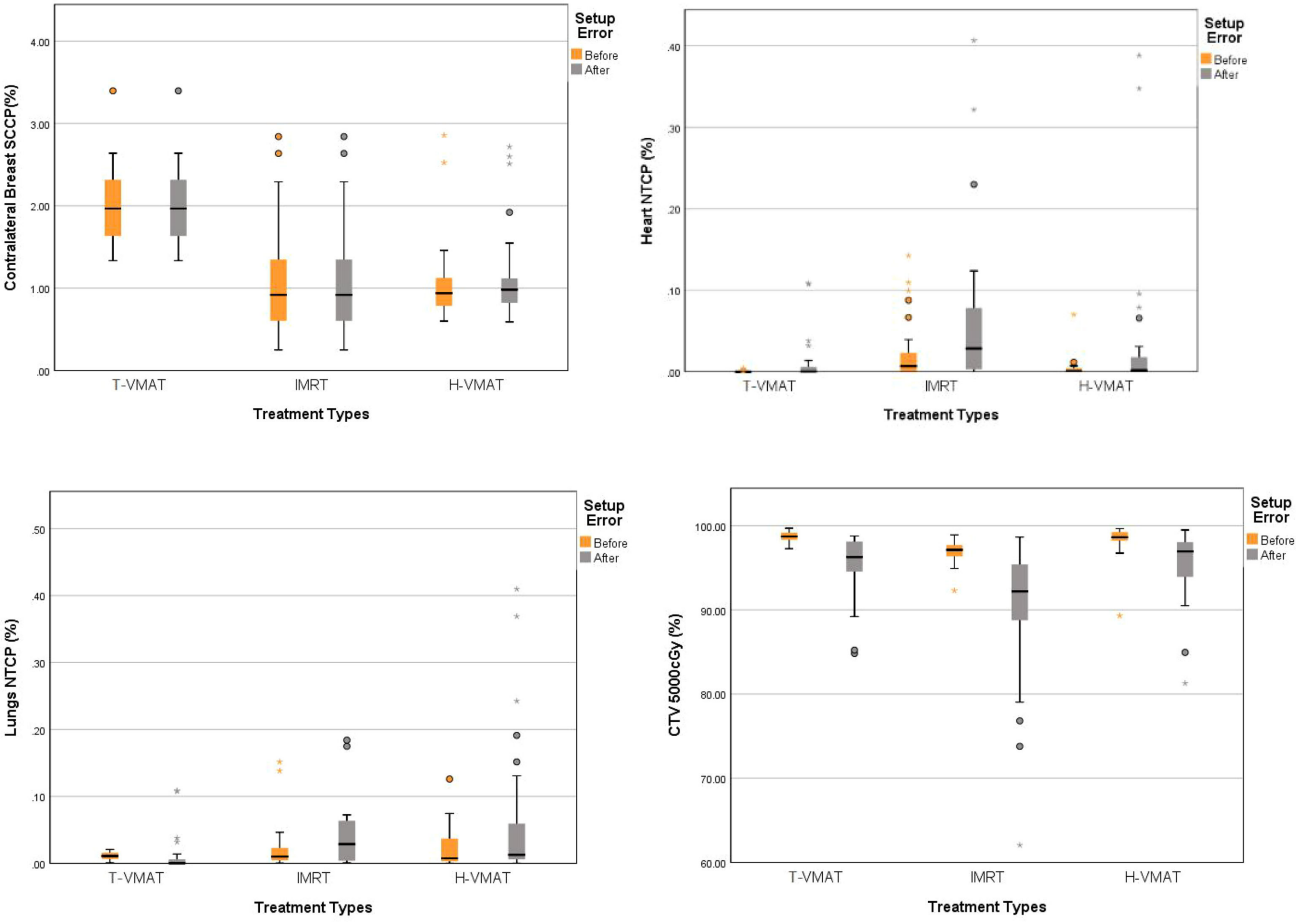
Box-whisker plot of mean radiobiological parameters with error bar: CTV coverage, Contralateral breast SCCP, Heart NTCP, Lungs NTCP with error bars. Each figures show the parameters changes of each organs before and after the recalculation of setup error. Gray bars represent the accumulated dose after the consideration of setup error, and yellow bars represent the dose not considered of setup error. The points in the graph represent outliers, the black horizontal lines in the figures represent the average of each parameter.

First, the NTCP-Poisson LQ was used to analyze mortality as the endpoint event of the cardiac biological model. The T-VMAT technique had the lowest NTCP before and after adding the setup error (0.0003% and 0.01%), and the highest NTCP was found after implementing the IMRT technique (0.21% and 0.2%). The pairwise comparison showed significant differences (P ≤ 0.001). The lung endpoint event was analyzed by the LKB model as radiation pneumonia ≥ level 2. The T-VMAT technique had an advantage. The NTCP values before and after adding the setup error were 0.01% and 0.024%, respectively, which were the lowest values among the three techniques. IMRT had the highest values (0.2% and 0.35%), and pairwise comparisons showed significant differences (P ≤ 0.001). For the SCCP of CB, the secondary incidence of tumors was the endpoint event. T-VMAT had significantly higher secondary incidences of CB than IMRT and H-VMAT, which were 2%, 1.05%, and 1.05%, respectively. After introducing the positioning error, the SCCP was still the highest for T-VMAT (2.01%). For H-VMAT and T-VMAT, the SCCP before and after adding the setting error was not significantly different (P ≤ 0.059 and P ≤ 0.185), but the comparison between IMRT and T-VMAT showed a significant difference (P ≤ 0.024). The biological model analysis was performed to determine significant differences and compare the advantages and disadvantages of various planning methods, as shown in [**Figure 6**]. All dosimetric parameters, biological indices, and the delivery efficiency are shown in **Table 3** and the p-values are shown in **Table 4**.

**Table 3:** Summary of the dosimetric parameters, radiobiological indices, and delivery parameters.

Structures	Metric	Conventional			After Setup Error		
		VMAT	IMRT	H-VMAT	VMAT	IMRT	H-VMAT
CTV	D5000cGy(%)	98.67 ± 0.6	96.97 ± 1.28	98.31 ± 1.75	95.51 ± 3.49	91.25 ± 7.83	95.48 ± 4.03
	V105(%)	53.86 ± 9.9	42.93 ± 1.28	49.5 ± 14.3	43.67 ± 12.62	37.31 ± 9.83	41.32 ± 15.09
	D2cc (cGy)	5423.28 ± 36.34	5482.17 ± 58.8	5421.8 ± 77.75	5425.52 ± 66.3	5504.42 ± 98.1	5462.63 ± 1215.7
Heart	V20(%)	5.34 ± 2.56	11.02 ± 4.25	12.39 ± 5.5	7.86 ± 4.25	12.97 ± 5.01	14.12 ± 6.16
	V30(%)	2.13 ± 1.44	7.15 ± 3.27	8.63 ± 4.21	3.75 ± 2.8	8.84 ± 4.09	10.03 ± 5.02
	D mean (cGy)	534.8 ± 108.3	672.58 ± 178.2	679.4 ± 175.63	626.3 ± 174.9	759.38 ± 226.1	763.22 ± 219.3
	NTCP (%)	.0003 ± .0008	.21 ± 0.06	.004 ± 0.012	.01 ± 0.02	.2 ± 1.04	.04 ± 0.09
LAD	Dmean (cGy)	2890.48 ± 769.4	3847.1 ± 883.9	3606.4 ± 792.4	3021.1 ± 1081.9	4000 ± 926.01	3666.84 ± 942.8
LV	Dmean (cGy)	576 ± 215.8	961.5 ± 246.1	980.97 ± 282.6	717.73 ± 166.2	1031.5 ± 261.2	1050 ± 288.02
Ipsilateral Lung	V5(%)	51.2 ± 3.8	52.95 ± 10.27	50.49 ± 6.11	52.47 ± 4.63	54.07 ± 10.3	52.7 ± 5.12
	V20(%)	22.2 ± 2.39	29.6 ± 4.7	28.81 ± 5.8	24.21 ± 3.58	32.2 ± 5.31	31.08 ± 5.50
	V30(%)	13.66 ± 1.7	21.75 ± 3.2	22.84 ± 5.28	16.67 ± 5.6	24.6 ± 3.73	25.08 ± 5.53
	Dmean (cGy)	1192.03 ± 91	1490.8 ± 171.5	1399.9 ± 212.1	1280.82 ± 149.3	1591.3 ± 206.1	1511.25 ± 213.04
Lungs	V5(%)	47.18 ± 5.73	27.52 ± 8.5	38.86 ± 5.488	48.10 ± 6.57	21.55 ± 10.1	40.05 ± 5.89
	V20(%)	11.4 ± 2.37	13.77 ± 3.95	13.03 ± 3.21	12.25 ± 3.04	14.01 ± 4.06	15.43 ± 5.01
	Dmean (cGy)	833.09 ± 98.12	761.12 ± 217.1	837.1 ± 124.06	892.49 ± 128.71	765.79 ± 208.2	881.68 ± 150.44
	NTCP (%)	.01 ± 0.005	.2 ± 0.904	.05 ± 0.18	.024 ± 0.054	.35 ± 1.0	.214 ± 22.35
Contralateral Lung	V5(%)	43.37 ± 11.75	6.64 ± 6.7	28.5 ± 8.3	45.4 ± 9.94	7.64 ± 7.48	30.17 ± 8.49
	V20(%)	2.77 ± 1.66	0.17 ± 0.4	0.18 ± 0.29	3.02 ± 1.77	0.21 ± 0.49	0.44 ± 1.14
	Dmean (cGy)	576.70 ± 112.6	161.07 ± 84.2	374.65 ± 78.22	592.6 ± 120.03	173.05 ± 91.31	394.88 ± 87.97
Contralateral Breast	V5(%)	74.14 ± 20.74	26.5 ± 21.6	11.62 ± 17.18	73.82 ± 20.44	28.33 ± 23.05	13.88 ± 22.46
	Dmean (cGy)	711.75 ± 160.14	357.6 ± 242.3	354.03 ± 1.05	699.54 ± 132.01	381.83 ± 242.9	381.77 ± 203.85
	SCCP (%)	2 ± 0.429	1.05 ± 0.65	1.049 ± 0.47	2.01 ± 0.43	1.06 ± 0.64	1.12 ± 0.55
MUs		746.25 ± 81.6	2098 ± 258.4	742.34 ± 69.6	–	–	–
TD Time(s)		168.62 ± 13.8	365.7 ± 29.8	169.5 ± 15.9	–	–	–
MF		3.73 ± 0.41	10.5 ± 1.29	3.71 ± 0.34	–	–	–

**Table 4:** P values for three techniques comparison using Wilcoxon signed-rank test.

Structures	Metric	Conventional P Value			After Setup Error P Value		
		VMAT VS IMRT	IMRT VS H-VMAT	VMAT VS H-VMAT	VMAT VS IMRT	IMRT VS H-VMAT	VMAT VS H-VMAT
CTV	D5000cGy(%)	≤.001**	≤.086	≤.001**	≤.001**	≤.001**	≤.428
	V105(%)	≤.611	≤.012*	≤.044*	≤.171	≤.019*	≤.324
	D2cc (cGy)	≤.009*	≤.012*	≤.001**	≤.029*	≤.001**	≤.001**
Heart	V20(%)	≤.006*	≤.152	≤.001**	≤.370	≤.256	≤.044*
	V30(%)	≤.001**	≤.166	≤.001**	≤.038*	≤.264	≤.002*
	D mean (cGy)	≤.007*	≤.936	≤.009*	≤.158	≤.867	≤.213
	NTCP (%)	≤.001**	≤.001**	≤.001**	≤.001**	≤.001**	≤.001**
LAD	Dmean (cGy)	≤.001**	≤.344	≤.138	≤.013*	≤.043*	≤.044*
LV	Dmean (cGy)	≤.001**	≤.445	≤. 092	≤.039*	≤.003*	≤. 871
Ipsilateral Lung	V5(%)	≤.001**	≤.005*	≤.011*	≤.001**	≤.001**	≤.582
	V20(%)	≤.001**	≤.287	≤.001**	≤.032*	≤.841	≤.019*
	V30(%)	≤.001**	≤.006*	≤.001**	≤.028*	≤.033*	≤.949
	Dmean (cGy)	≤.001**	≤.244	≤.001**	≤.077	≤.858	≤.052
Lungs	V5(%)	≤.032*	≤.017*	≤.809	≤.001**	≤.001**	≤.543
	V20(%)	≤.006*	≤.251	≤.100	≤.112	≤.247	≤.007*
	Dmean (cGy)	≤.001**	≤.003*	≤.197	≤.009*	≤.076	≤.390
	NTCP (%)	≤.001**	≤.001**	≤.001**	≤.001**	≤.001**	≤.001**
Contralateral Lung	V5(%)	≤.002*	≤.241	≤.006*	≤.119	≤.487	≤.383
	V20(%)	≤.001**	≤.045*	≤.001**	≤.001**	≤.001**	≤.017*
	Dmean (cGy)	≤.110	≤.685	≤.046*	≤.133	≤.836	≤.088
Contralateral Breast	V5(%)	≤.817	≤.205	≤.046*	≤.508	≤.836	≤.605
	Dmean (cGy)	≤.024*	≤.081	≤.059	≤.001**	≤.334	≤.018*
	SCCP (%)	≤.024*	≤.081	≤.059	≤.024*	≤.340	≤.185
MUs		≤.001**	≤.001**	≤.379	–	–	–
TD Time(s)		≤.001**	≤.001**	≤.873	–	–	–
MF		≤.001**	≤.001**	≤.388	–	–	–

^*^The P-value is lower than 0.05.

^**^ The P-value is lower than 0.001.

### Delivery Parameters and Plan Complexity

Regarding the delivery parameters (25), IMRT had the highest treatment MUs (2,098) and total delivery time (365.7 s). The values were considerably higher than those of T-VMAT (746.25 and 168 s) and H-VMAT (the 742.34 and 169.5 s). The results of the analysis of plan complexity using modulation factor (26, 27) showed that T-VMAT and H-VMAT also had significantly lesser values than those of IMRT, which were 3.73, 3.71, and 10.5, respectively. IMRT and the other two treatment techniques showed significant differences in the delivery time, MUs, and treatment difficulty (P ≤ 0.001).

## Discussion

For more accurate planning, the quality of the plan needs to be better, and the robustness and complexity of the plan need to be analyzed quantitatively. In 2020, a study (25) suggested that the dose distribution was not similar to the dose delivered to the patient due to uncertainties in dose calculation and treatment delivery, including variations in patient setup and anatomy. C-RAD systems can quantify setup errors in PMRT for breast cancer treatment. In our traditional radiation therapy, CBCT was a key method for assessing the positioning error (11, 26). It enabled us to visualize important anatomical details in the patient’s body. Additionally, many recent studies have shown that optical body surface monitoring can also assess the patient’s setup error, especially in breast cancer patients (28, 29). Theoretically, the target volume is closer to the chest wall for PMRT patients than the patients undergoing breast-conserving therapy, which makes the effect of SGRT more robust and accurate. The dose accumulation obtained by SGRT can extract real-time isocenter shifts, which has great advantages for analyzing intrafraction errors (30). Therefore, in this study, the registered body surface image after performing CBCT was used as the reference. The optical body surface image obtained before treatment and the optical body surface data recorded during the treatment were used to analyze the intra-fraction error. The superposition of the two setup errors was used for dose accumulation to obtain the real-world dose distribution. The non-rigid registration algorithm was selected for image registration. Because each part of ROI was given a corresponding weight according to the distance from the isocenter, a slightly larger or smaller ROI had negligible effects on the results. The rigid algorithm superimposed the changes of skin folds and other changes in the ROI to the final result regardless of the severity, and thus, we used non-rigid registration more in clinical analysis.

The radiobiological response model was used to compare the advantages and disadvantages of the different techniques (7, 8, 31). In general, no technique was better than the other two techniques in all standards, in our dose analysis, after accounting for the setup error. Although the dose advantage of the T-VMAT technique for the lungs and heart was prominent, the dose for CB in T-VMAT was significantly higher than the dose in IMRT and H-VMAT. Stovall et al. described the effect of dose on CB of breast cancer patients. They found that younger patients were more likely to have a long-term risk of breast cancer (32). Macduff et al. evaluated patients below 45 years who were carrying certain rare ATM variants, and they should be more aware of the risk of SCCP of CB cancer (33). This led us to analyze the secondary incidence of breast cancer using the T-VMAT technique, and the SCCP of CB was significantly higher than that in the other two techniques. In the analysis of biological models, the lung and heart complication rates of IMRT were the highest, while the clinical target area coverage was the least. This indicated that, based on setting errors, the impact received was the greatest, which made IMRT the least favored technique in this study. The situation concerning H-VMAT was different. After dose accumulation, the NTCP of the heart and lungs for H-VMAT was significantly lower than that for IMRT and slightly higher than that for T-VMAT. Thus, the dosimetry parameters and the probability of complications met the clinical conditions and standards. Moreover, H-VMAT had a small impact on setup errors and covered a higher proportion of CTV after introducing the positioning errors. This might immensely help to control the local rate after breast cancer surgery. Thus, H-VMAT can be used clinically in PMRT patients with internal mammary lymph nodes to achieve target dose coverage; additionally, the OAR dose and NTCP were found to be relatively well-balanced.

Among the limitations of the study, the intrafraction error of the optical body surface images for each treatment field in SGRT was not precise. Every patient was treated using a specific treatment technique, it was impossible to obtain the three intrafraction setup errors in one patient. Thus, we selected 10 patients per technique to estimate each intrafraction setup error during treatment. To ensure greater accuracy, we only extracted patient data from fields with the same angles. The interfraction error of each time and the intrafraction error of each field were only approximated to the actual error. CBCT-based image registration is the most recognized method because anatomical structures can be seen, and tumor changes within the target volume are always visible. In this study, PMRT was used to treat patients who had no tumor tissue in the planning target, and the tumor location was close to the optical body surface, and thus, the impact could be minimized. Implementing adaptive radiotherapy (ART) might solve this problem (11). Additionally, the rotation of the patient in two directions (Pitch and Roll) were not accounted for. We wanted to simulate the scenarios introduced by the isocenter to set uncertainty, improve the accuracy of dosimetry, determine the robustness and complexity of the plan, and calculate the bioequivalent dose based on dosimetry. For the whole process, an overall analysis from plan design and implementation to prognosis was conducted. Moreover, the deep inspiration breath-hold (DIBH) technology can significantly reduce the radiation dose that the heart and lungs are exposed to during breast cancer radiotherapy (34, 35). However, the treatment involving free breathing is still the conventional procedure for treating PMRT patients at our center. Hence, the DIBH technique was not used in this study. Future studies can combine various techniques with the DIBH technique for PMRT (15, 36).

## Conclusion

H-VMAT technique can provide an appropriate balance of target coverage, OAR dose, complication probability, planning of robustness, and delivery efficiency relative to IMRT and VMAT techniques in PMRT patients with internal mammary lymph nodes. We propose a method using SGRT to evaluate the impact of different planning modalities on setup error, which reflected the robustness of the plan in the plan design. In the future, the robustness and complexity of the plan need to be quantified, and the long-term clinical outcomes have to be evaluated to assess its reliability.

## Data Availability Statement

The raw data supporting the conclusions of this article will be made available by the authors, without undue reservation.

## Author Contributions

ZZ conceived idea and wrote the manuscript. PY, ZP, and XL helped with programming and DL analyzed data, XQ and WS helped with statistical analysis. FP and ZT helped with editing the manuscript. YL and YW checked results and critically revised the manuscript. All authors contributed to the article and approved the submitted version.

## Conflict of Interest

The authors declare that the research was conducted in the absence of any commercial or financial relationships that could be construed as a potential conflict of interest.

## Publisher’s Note

All claims expressed in this article are solely those of the authors and do not necessarily represent those of their affiliated organizations, or those of the publisher, the editors and the reviewers. Any product that may be evaluated in this article, or claim that may be made by its manufacturer, is not guaranteed or endorsed by the publisher.

## Abbreviations

LBC, left-sided breast cancer; PMRT, postmastectomy radiotherapy; IMNI, internal lymph mammary nodes irradiation; L, ipsilateral lung; CB, contralateral breast; PTV, planning target volume; IMRT, Intensity-modulated radiotherapy; T-VMAT, Tangential volumetric modulated arc therapy; H-VMAT, Hybrid VMAT; MUs, monitor units; CT, computed tomography; DVH, dose-volume histogram; SGRT, surface gated radiotherapy DIBH, deep inspiration breath hold; TD-TIME, Total delivery time; LAD, left anterior descending artery; LV, left ventricle; NTCP, normal tissue complication probability; SCCP, second cancer complication probability; CBCT, Cone-beam computed tomography.
